# Left atrial passive function after aortic valve replacement in aortic stenosis

**DOI:** 10.1186/1532-429X-16-S1-P237

**Published:** 2014-01-16

**Authors:** Hoshang Farhad, Tomas Neilan, Siddique Abbasi, Ravi V Shah, Jiazuo Feng, Raymond Y Kwong, Michael Jerosch-Herold

**Affiliations:** 1 Cardiovascular Division, Department of Medicine, Brigham and Women's Hospital, Boston, Massachusetts, USA; 2 Cardiac MR PET CT Program, Department of Radiology, Massachusetts General Hospital, Boston, Massachusetts, USA; 3 Division of Cardiology, Department of Medicine, Massachusetts General Hospital, Boston, Massachusetts, USA; 4 Department of Radiology, Brigham and Women's Hospital, Harvard Medical School, Boston, Massachusetts, USA

## Background

Aortic valve replacement (AVR) is the definitive treatment for severe symptomatic aortic stenosis (AS). Aortic stenosis is associated with diastolic dysfunction and left atrial (LA) enlargement. After a successful AVR, there is a decrease in LA size but persistence in diatolic dysfunction. We hypothesized that LA function would help link this discordance. Cardiac magnetic resonance (CMR) is the gold standard for assessment of the LA. We therefore aimed to test the effect of AS on LA function and the subsequent effects of an AVR on LA function. We hypothesized that, similar to diastolic function, LA function would not improve post-AVR and that the persistence in LA dysfunction might be related to expansion of the extracellular space.

## Methods

A comprehensive CMR exam was performed on 18 patients with isolated AS and without coronary disease pre- and 1 year post-AVR. Results were compared to age- and gender matched healthy controls. Left atrial volumes (LAV) were calculated at the end of ventricular systole (LAVmax), just before atrial contraction (LAVbac), and at the end of ventricular diastole (LAVmin) using the biplane area-length method. Left atrial passive emptying fraction (LAPEF) defined by (LAVmax-LAVbac) × 100/LAVmax, as well as left atrial contractile emptying fraction (LACEF) defined by (LAVbac-LAVmin) × 100/LAVbac were calculated. T1 measurements were made in the myocardium and blood before and after contrast administration using a Look-Locker sequence with a gradient echo cine acquisition. The ECV was calculated by comparing the change in the R1 values from blood to myocardium and integrating the hematocrit.

## Results

Patients were predominantly male (67%) with a mean age of 61 ± 12 years, and a mean LVEF of 62 ± 5%. Prior to AVR, patients with AS had an increased left ventricular (LV) mass, increased LA volume, reduced LAPEF, and an increased ECV (Table 1). At one year after AVR, there was a marked reduction in LV mass and a decrease in LA volume. However, there was further impairment in LAPEF and a continued increase in the ECV at 1 year post-AVR (Table, Graph). There was a strong inverse association between the LAPEF and the ECV (r = -0.70, p < 0.001) and a strong inverse association between the decline in LAPEF and the increase in the ECV post AVR (r = -0.71, p < 0.001).

**Table 1:** CMR data in Healthy Controls and Patients with Severe AS pre- and post-AVR

Variable	Healthy Controls (N = 6)*	AS Pre-AVR (N = 18)* **	AS Post-AVR (N = 18) **	P-value, ANOVA	*P-value, Healthy Controls vs. AS Pre-AVR	**P-value, AS Pre-AVR vs. AS Post-AVR
Age (years)	60 ± 8	60 ± 11	62 ± 10	0.91	1.00	0.29

Male (%)	66.67	66.67	66.67	1.00	1.00	1.00

Systolic Blood Pressure (mmHg)	120 ± 4	123 ± 9	134 ± 12	0.001	0.56	0.0004

Diastolic Blood Pressure (mmHg)	74 ± 7	79 ± 8	76 ± 12	0.38	0.17	0.16

Heart rate (beats/min)	72 ± 12	68 ± 10	68 ± 10	0.61	0.35	0.85

BMI (kg/m2)	25 ± 6	28 ± 6	28 ± 6	0.55	0.26	0.16

Cardiac Magnetic Resonance:						

LV EF (%)	64 ± 5	67 ± 7	61 ± 5	0.01	0.21	0.005

LVEDV (mls)	123 ± 21	147 ± 38	132 ± 25	0.19	0.14	0.03

LVESV (mls)	43 ± 13	49 ± 19	51 ± 13	0.72	0.65	0.6

LV mass index (g/m2)	47 ± 5	72 ± 12	60 ± 8	<0.001	<0.0001	0.0003

RVEF (%)	53.2 ± 2	58.9 ± 7.2	54.8 ± 5.7	0.063	0.07	0.005

ECV	0.28 ± 0.03	0.33 ± 0.04	0.36 ± 0.03	<0.001	0.01	<0.001

LAV max index (ml/m2)	31 ± 8	50 ± 14	34 ± 9	<0.001	0.003	0.0009

LAV bac index (ml/m2)	19 ± 7	37 ± 12	30 ± 7	<0.001	0.001	0.03

LAV min index (ml/m2)	12 ± 5	20 ± 6	17 ± 4	0.009	0.009	0.22

LAPEF (%)	40 ± 9	26 ± 8	13 ± 9	<0.001	0.003	<0.0001

LACEF (%)	39 ± 5.2	46 ± 12	41 ± 9	0.26	0.18	0.3

## Conclusions

Severe AS is associated with a reduction in LA passive function. After AVR, LAPEF continues to decline and there was a strong inverse association between LAPEF and the ECV.

## Funding

Dr. Neilan is supported by an American Heart Association Fellow to Faculty Grant (12FTF12060588). Dr. Jerosch-Herold is supported in part by a research grant from the National Institutes of Health (R01HL 090634-01A1).

**Figure 1:**
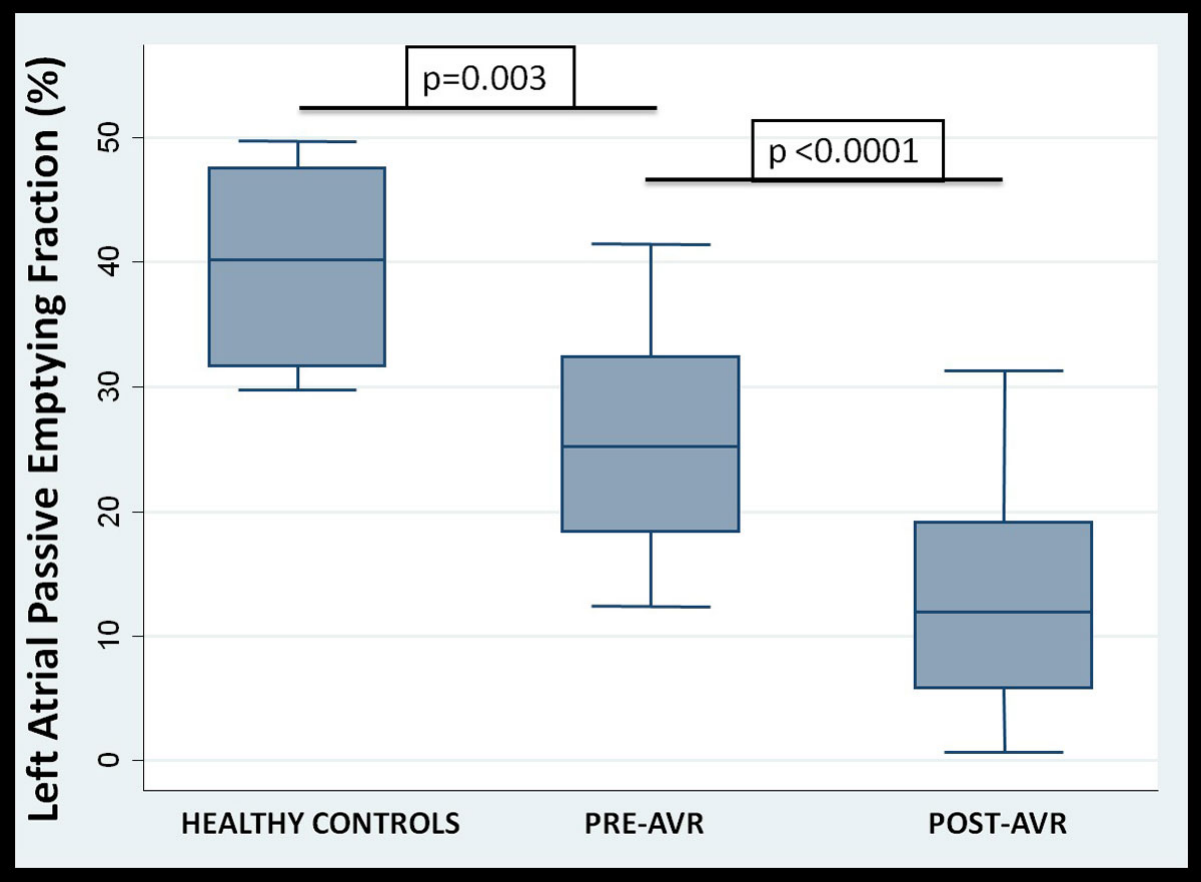
**Box plot of Left Atrial Passive Emptying Fraction among healthy controls, and patients with severe AS pre- and post-AVR Graph: Left atrial passive emptying fraction (LAPEF) among healthy controls, and patients with AS, pre-and post-AVR showing a reduction in LAPEF pre-AVR and a further decline in LAPEF at one year post-AVR**.

